# Acute and long-term renal effects after iodine contrast media–enhanced computerised tomography in the critically ill—a retrospective bi-centre cohort study

**DOI:** 10.1007/s00330-023-10059-7

**Published:** 2023-09-02

**Authors:** Felix Berglund, Ebba Eilertz, Fredrik Nimmersjö, Adam Wolf, Christopher Nordlander, Fredrik Palm, Fredric Parenmark, Johan Westerbergh, Per Liss, Robert Frithiof

**Affiliations:** 1 Department of Surgical Sciences, Radiology, Uppsala University, Uppsala, Sweden; 2 Department of Surgical Sciences, Uppsala University, Uppsala, Sweden; 3 Department of Medical Cell Biology, Uppsala University, Uppsala, Sweden; 4 Centre for Research and Development, Uppsala University, Uppsala, Sweden; 5 Uppsala Clinical Research Center, Uppsala University, Uppsala, Sweden; 6 Department of Surgical Sciences, Anesthesiology and Intensive Care, Uppsala University, Uppsala, Sweden

**Keywords:** Critical care, Contrast media, Acute kidney injury, Tomography, X-ray computed

## Abstract

**Objectives:**

To determine if current clinical use of iodine contrast media (ICM) for computerised tomography (CT) increases the risk of acute kidney injury (AKI) and long-term decline in renal function in patients treated in intensive care.

**Methods:**

A retrospective bi-centre cohort study was performed with critically ill subjects undergoing either ICM-enhanced or unenhanced CT. AKI was defined and staged based on the Kidney Disease Improve Global Outcome AKI criteria, using both creatinine and urine output criteria. Follow-up plasma creatinine was recorded three to six months after CT to assess any long-term effects of ICM on renal function.

**Results:**

In total, 611 patients were included in the final analysis, median age was 65.0 years (48.0–73.0, quartile 1–quartile 3 (IQR)) and 62.5% were male. Renal replacement therapy was used post-CT in 12.9% and 180-day mortality was 31.2%. Plasma creatinine level on day of CT was 100.0 µmol/L (66.0–166.5, IQR) for non-ICM group and 77.0 µmol/L (59.0–109.0, IQR) for the ICM group. The adjusted odds ratio for developing AKI if the patient received ICM was 1.03 (95% confidence interval 0.64–1.66, *p* = 0.90). No significant association between ICM and increase in plasma creatinine at long-term follow-up was found, with an adjusted effect size of 2.92 (95% confidence interval − 6.52–12.36, *p* = 0.543).

**Conclusions:**

The results of this study do not indicate an increased risk of AKI or long-term decline in renal function when ICM is used for enhanced CT in patients treated at intensive care units.

**Clinical relevance statement:**

Patients treated in intensive care units had no increased risk of acute kidney injury or persistent decline in renal function after contrast-enhanced CT. This information underlines the need for a proper risk-reward assessment before denying patients a contrast-enhanced CT.

**Key Points:**

• *Iodine contrast media is considered a risk factor for the development of acute kidney injury*.

• *Patients receiving iodine contrast media did not have an increased incidence of acute kidney injury or persistent decline in renal function*.

• *A more clearly defined risk of iodine contrast media helps guide clinical decisions whether to perform contrast-enhanced CTs or not*.

**Supplementary information:**

The online version contains supplementary material available at 10.1007/s00330-023-10059-7.

## Introduction

Acute kidney injury (AKI) is a common complication in patients treated in intensive care units [1]. AKI develops in more than 50% of patients in intensive care and is associated with increased length of stay, need for renal replacement therapy (RRT), progression of chronic kidney disease (CKD), and increased mortality [2, 3]. Patients receiving iodine contrast media (ICM) are often considered to have an increased risk of AKI [1].

Post-contrast acute kidney injury (PC-AKI) is any AKI that occurs within 48–72 h following ICM exposure [4, 5]. The impact of PC-AKI on critically ill patients is unclear and the reported incidence ranges from 7 to 22% [6–8]. Though ICM-enhanced computerised tomography (CT) is an invaluable diagnostic tool for common conditions in intensive care units (ICUs), its use is weighed against the risk of PC-AKI, which is not clearly defined. Investigations with propensity score–matched control groups have not found any clinically meaningful toxicity of ICM in the critically ill [8, 9]. ICM’s role as a risk factor for AKI has been questioned in other patient cohorts as well [10–16]. Furthermore, attributing a decline in renal function to ICM should not be done without a proper control group, since reduced renal function often develops in unexposed ICU patients as well [9].

The association between ICM and AKI in critically ill patients is currently not clarified. In everyday clinical practice, this leads to discussions of acute benefits versus risks of AKI prior to ICM-enhanced radiological examinations. Furthermore, since there is a lack of long-term data, it is unclear whether PC-AKI causes any relevant long-term complications.

The aim of this study was to investigate if ICM is associated with an increased incidence of AKI or long-term decline in renal function in critically ill patients. We hypothesised that ICM, in the form currently used to enhance CT, does not increase the risk of AKI or long-term decline in renal function.

## Materials and methods

This study was approved by the Swedish Ethical Review Authority (Dnr 2017/168 with amendment 2020–00135). Declaration of Helsinki and its subsequent revisions were observed.

This was a retrospective bi-centre cohort study performed at a tertiary university hospital and a secondary county hospital. STROBE guidelines were followed for reporting [17].

Critically ill patients were defined as patients treated in the intensive care unit at the time the CT was requested and performed. All patients referred from the general ICU for a CT at the university hospital between January 2013 and February 2020, or at the county hospital from January 2015 to December 2017, were eligible for inclusion. Whether ICM was used or not was guided by the patients’ needs and current guidelines [18]. The following exclusion criteria were used:

- ICU treatment < 48 h
- RRT before CT
- No plasma creatinine value for CT date or any of the three following days
- Age < 16 years

Background data, clinical history, the main reason for ICU admission, development of acute heart failure or acute liver failure during the ICU stay, and Simplified Acute Physiology Score 3 (SAPS 3) were documented [19]. Details regarding CT included if ICM was used and, if so, which dose and type. Treatments received included mechanical respiratory treatments (divided into invasive and non-invasive), antibiotics, vasoactive drugs, and drugs affecting the kidneys. Any use of these drugs within 24 h pre- or post-CT, except diuretics, where the time limit was extended to 48 h post-CT, was recorded. Treatment with N-acetylcysteine before CT was also noted. Physiological parameters and fluid treatment were also documented.

Plasma creatinine values were recorded at seven different occasions. Values were logged three months before CT or earlier (referred to as the patient’s baseline value), on day of ICU admission, on day of CT, daily on the first 3 days following CT, and at least and as close to three months after CT as possible, but no later than six months after CT, for long-term follow-up. The revised Lund-Malmö formula and plasma creatinine levels were used to calculate the estimated glomerular filtration rate (eGFR) [20]. If the baseline plasma creatinine value was missing, it was estimated using the Modification of Diet in Renal Disease (MDRD) formula and a set eGFR of 75 mL/min/1.73 m^2^ [21]. Total urine output was measured once daily for the three days following CT.

The primary outcome, AKI, was defined and staged in accordance with the Kidney Disease Improve Global Outcome (KDIGO) criteria [22]. The baseline plasma creatinine value was compared with the values from the 3 days following CT. Sensitivity analysis included if the patients developed PC-AKI, defined as the development of AKI within 72 h of ICM administration, with plasma creatinine value on day of CT used as a baseline. For patients not receiving ICM, PC-AKI should be read as post-CT AKI. The plasma creatinine value on day of CT was compared with the baseline value, to determine if the patient fulfilled AKI criteria before ICM was given. A second sensitivity analysis was performed where all patients who had additional CTs during the 72 h following their first exam were removed. An additional sensitivity analysis was if the patients had a > 25% increase in plasma creatinine either during the 72 h following CT compared with plasma creatinine value on day of CT or during the 72 h following CT compared with any of the preceding days during those 72 h. RRT and mortality post-CT were also documented.

All statistical analyses were made using R (version 3.6.1). For tests of differences among groups, the chi-squared test was used for categorical variables and the Kruskal–Wallis’s test for continuous variables. Association with outcome was evaluated using logistic regression. All statistical tests were two-tailed, and a 0.05 significance level was used. Imputation was performed where data were missing using the package *mice*. Further details regarding which parameters were collected, sensitivity analysis, the statistical analyses made, and models used can be found in Supplement A.

## Results

**Fig. 1:**
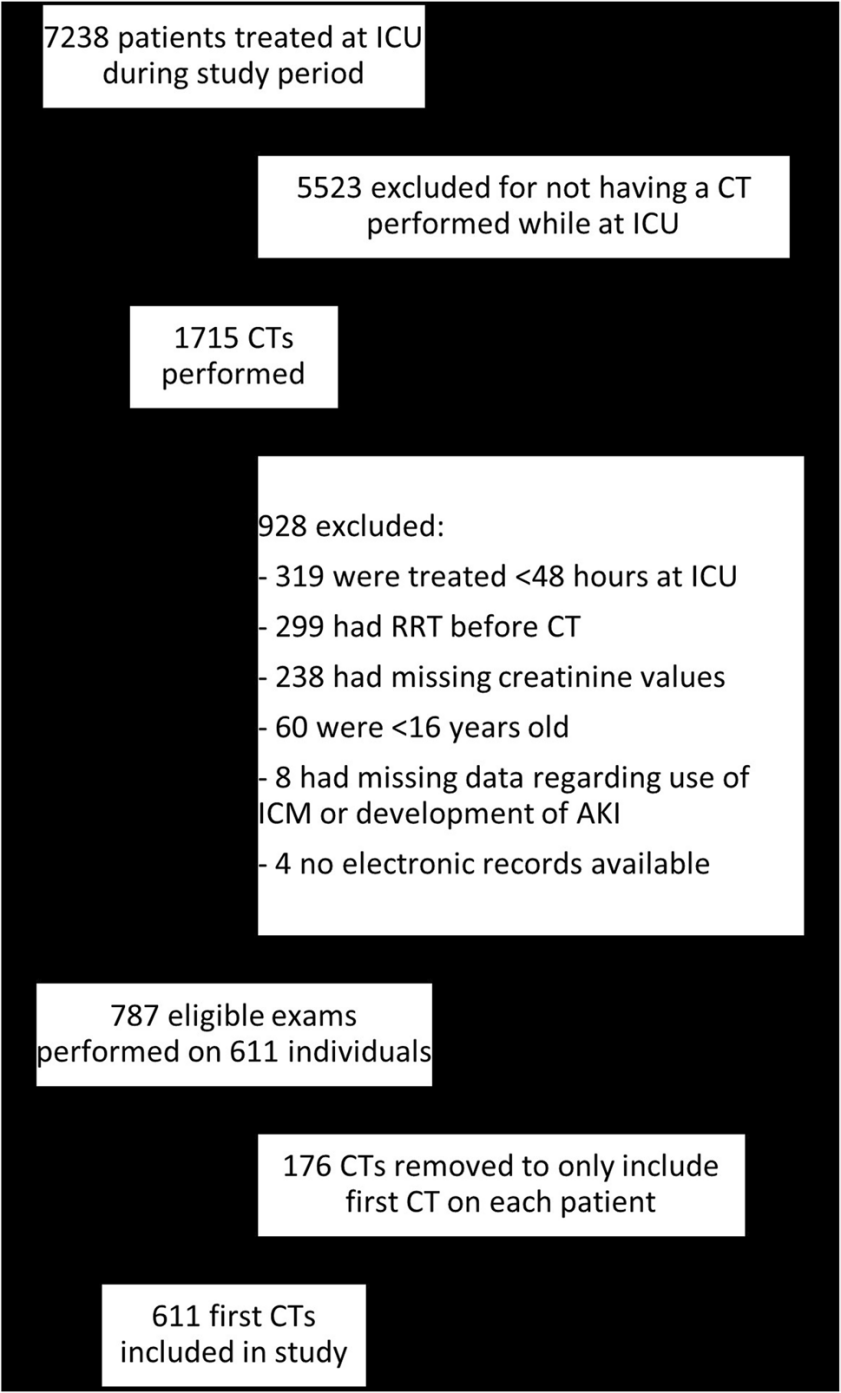
Study inclusion flowchart

During the study period, 1715 CTs were performed: 1415 at the university hospital and 300 at the county hospital. After applying exclusion criteria, 787 CTs on 611 individuals remained. Of these 611 individuals, 114 had two or more exams. Since only the first CTs was used in the analysis, 611 exams on 611 individuals could be analysed (Fig. 1). Of these, 264 were performed with ICM and 347 without. In total, 506 of the CTs were performed at the larger university hospital and 105 at the county hospital. All patients had an acute indication of having their CT.

The groups did not differ in sex distribution. The ICM group had a lower prevalence of AKI compared with baseline on day of CT as well as lower plasma creatinine values both at baseline, on day of CT, and at follow-up. They also had a lower prevalence of CKD and a lower SAPS 3. An equal proportion of patients had their baseline plasma creatinine estimated in both groups. The groups did not differ significantly in the use of renal-affecting or vasoactive drugs (Table 1). The most used ICM was Iohexol 350 mg I/mL used in a dose of 1.0 mL/kg of body weight (Table S1), and the most examined body part was the head for patients not receiving ICM and the thorax for patients receiving ICM (Table S2).

**Table 1:** Patient characteristics

	*n*	Combined *n* = 611	Contrast at CT		*p* value
			No contrast *n* = 347	Contrast *n* = 264	
Demography					
Hospital: University hospital	611	506 (82.8%)	288 (83.0%)	218 (82.6%)	0.91^1^
County hospital		105 (17.2%)	59 (17.0%)	46 (17.4%)	
Age	610	65.0 (48.0–73.0) [1]	66.0 (51.0–73.0) [1]	62.0 (43.8–73.0) [0]	0.071^2^
Sex: Men	611	382 (62.5%)	219 (63.1%)	163 (61.7%)	0.74^1^
Women		229 (37.5%)	128 (36.9%)	101 (38.3%)	
Cause of admission					
Circulatory failure	609	31 (5.1%) [2]	22 (6.3%) [0]	9 (3.4%) [2]	
Cardiac arrest		58 (9.5%)	38 (11.0%)	20 (7.6%)	
Infection		129 (21.2%)	77 (22.2%)	52 (19.8%)	
Respiratory failure		169 (27.8%)	82 (23.6%)	87 (33.2%)	
Trauma		108 (17.7%)	62 (17.9%)	46 (17.6%)	
Other		114 (18.7%)	66 (19.0%)	48 (18.3%)	
Medical history					
Chronic kidney disease	572	39 (6.8%) [39]	33 (10.1%) [20]	6 (2.4%) [19]	< 0.001^1^
Heart failure	572	68 (11.9%) [39]	41 (12.6%) [22]	27 (10.9%) [17]	0.60^1^
Liver failure	572	25 (4.4%) [39]	16 (4.9%) [22]	9 (3.6%) [17]	0.54^1^
Hypertension	609	237 (38.9%) [2]	145 (41.9%) [1]	92 (35.0%) [1]	0.093^1^
Diabetes mellitus	610	117 (19.2%) [1]	73 (21.0%) [0]	44 (16.7%) [1]	0.21^1^
Medical events and clinical markers					
Simplified Acute Physiology Score 3	610	64.0 (55.0–73.0) [1]	65.0 (55.5–74.0) [0]	62.0 (54.0–71.0) [1]	0.035^2^
Highest lactate level	546	2.9 (1.9–4.4) [65]	3.0 (2.0–4.5) [43]	2.8 (1.9–4.3) [22]	0.24^2^
Lowest Hb	565	85.0 (78.0–97.0) [46]	84.0 (78.0–98.0) [25]	85.0 (79.0–96.0) [21]	0.75^2^
Mean arterial pressure at day of CT	575	75.0 (65.0–85.0) [36]	75.0 (65.0–84.0) [34]	76.0 (65.0–85.8) [2]	0.19^2^
Partial pressure of arterial oxygen to fractional inspired oxygen at CT	567	26.1 (18.9–36.0) [44]	26.2 (19.5–36.5) [33]	26.1 (18.2–36.0) [11]	0.45^2^
Days at intensive care unit	506	7.0 (4.0–12.0) [105]	6.0 (4.0–12.0) [59]	7.0 (4.0–13.0) [46]	0.44^2^
Acute kidney injury (AKI)					
AKI stage at day of CT: 0	611	399 (65.3%)	202 (58.2%)	197 (74.6%)	< 0.001^1^
1		117 (19.1%)	69 (19.9%)	48 (18.2%)	
2		58 (9.5%)	44 (12.7%)	14 (5.3%)	
3		37 (6.1%)	32 (9.2%)	5 (1.9%)	
Creatinine					
Baseline creatinine	611	84.0 (70.0–94.5) [2]	86.8 (70.9–97.5) [1]	78.0 (69.4–92.5) [1]	< 0.001^2^
Creatinine at day of CT	611	85.0 (62.0–132.0)	100.0 (66.0–166.5)	77.0 (59.0–109.0)	< 0.001^2^
Creatinine follow-up	298	71.5 (58.0–90.0) [313]	76.0 (63.0–96.0) [169]	67.0 (53.8–82.0) [144]	0.001^2^
Baseline creatinine estimated	611	228 (37.3%)	130 (37.5%)	98 (37.1%)	1.00^1^
eGFR baseline excluding estimated	292	74.0 (61.0–90.0) [319]	69.0 (58.5–84.0) [184]	81.0 (66.0–90.0) [135]	< 0.001^2^
Medication					
Use of renal-affecting drugs	592	447 (75.5%) [19]	250 (76.0%) [18]	197 (74.9%) [1]	0.77^1^
Number of renal-affecting drugs: 0	479	38 (7.9%) [132]	20 (7.5%) [80]	18 (8.5%) [52]	0.91^1^
1		332 (69.3%)	186 (69.7%)	146 (68.9%)	
2		97 (20.3%)	53 (19.9%)	44 (20.8%)	
3		10 (2.1%)	7 (2.6%)	3 (1.4%)	
4		2 (0.4%)	1 (0.4%)	1 (0.5%)	
Use of vasoactive drugs	602	489 (81.2%) [9]	267 (79.0%) [9]	222 (84.1%) [0]	0.12^1^
Number of vasoactive drugs: 0	514	28 (5.4%) [97]	16 (5.7%) [67]	12 (5.1%) [30]	0.59^1^
1		352 (68.5%)	193 (68.9%)	159 (67.9%)	
2		107 (20.8%)	60 (21.4%)	47 (20.1%)	
3		26 (5.1%)	11 (3.9%)	15 (6.4%)	
4		1 (0.2%)	0 (0.0%)	1 (0.4%)	
Ventilation					
Mechanical ventilation	609	568 (93.3%) [2]	318 (92.2%) [2]	250 (94.7%) [0]	0.25^1^
Type of ventilation: Invasive	407	343 (84.3%) [204]	199 (84.3%) [111]	144 (84.2%) [93]	0.22^1^
Non-invasive		23 (5.7%)	10 (4.2%)	13 (7.6%)	
No mechanical		41 (10.1%)	27 (11.4%)	14 (8.2%)	

*m* (*a*–*b*) represents the median (IQR)

*n* (*p*%) represents the frequency (percentage). Percentages computed by group

[*M*] represents the number of missing. Tests used: ^1^ Fisher’s exact test; ^2^ Wilcoxon test

The unadjusted model for the association between ICM at CT and AKI demonstrated a significant association with an odds ratio of 0.53 (95% CI 0.38–0.74, *p* ≤ 0.001), indicating a higher risk for AKI when ICM is not administered. However, when adjusted for confounding factors known to increase the risk of AKI, the odds ratio increased to 1.03 (95% CI 0.64–1.66) and no significant association between AKI and ICM remained (*p* = 0.90).

**Fig. 2:**
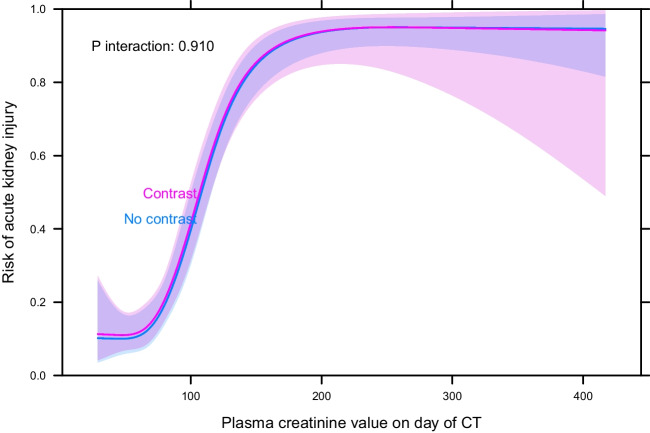
Predicted risk of developing AKI by plasma creatinine value (µmol/L) on day of CT for ICM vs. no ICM, unadjusted model (adjusted model yielded similar results). The figure shows the patient’s plasma creatinine value on day of CT on the X axis and the predicted risk of developing AKI on the Y axis; the shaded area represents the 95% confidence bands for the lines

There was no difference in the predicted risk of developing AKI for corresponding plasma creatinine values on day of CT (Fig. 2). Follow-up plasma creatinine values existed for 298 patients and long-term plasma creatinine was not found to be significantly affected by ICM. The unadjusted model demonstrated an effect size of − 5.20 (− 15.42–5.03, *p* = 0.318), while the adjusted model showed an effect size of 2.92 (− 6.52–12.36, *p* = 0.543).

The incidence of AKI following CT, defined by the combination of creatinine and urine output criteria, was 41.9%. PC-AKI incidence was 16.9% when defined only by creatinine criteria and 25.6% when both creatinine and urine output criteria were used (Table 2). The unadjusted odds ratio between the association of ICM at CT and PC-AKI was 0.67 (95% CI 0.46–0.97, *p* = 0.035); in the adjusted model, it increased to 0.94 (95% CI 0.61–1.43, *p* = 0.767). There was no difference in the predicted risk of developing PC-AKI for corresponding plasma creatinine values on day of CT, regardless of ICM exposure (Figure S1).

**Table 2:** Incidence and stage of AKI and PC-AKI. PC-AKI shown both as only defined by creatinine-based criteria and defined by all KDIGO criteria

	*n*	Combined *n* = 611	Contrast at CT	
			No contrast *n* = 347	Contrast *n* = 264
Acute kidney injury (AKI)				
AKI within 3 days: No	611	355 (58.1%)	179 (51.6%)	176 (66.7%)
Yes		256 (41.9%)	168 (48.4%)	88 (33.3%)
Highest stage of AKI days 1–3: 0	609	353 (58.0%) [2]	179 (51.6%) [0]	174 (66.4%) [2]
1		96 (15.8%)	57 (16.4%)	39 (14.9%)
2		81 (13.3%)	58 (16.7%)	23 (8.8%)
3		79 (13.0%)	53 (15.3%)	26 (9.9%)
Post-contrast acute kidney injury (PC-AKI): Creatinine criteria				
PC-AKI creatinine: No	610	507 (83.1%) [1]	282 (81.3%) [0]	225 (85.6%) [1]
Yes		103 (16.9%)	65 (18.7%)	38 (14.4%)
Highest stage of PC-AKI days 1–3: 0	610	507 (83.1%) [1]	282 (81.3%) [0]	225 (85.6%) [1]
1		83 (13.6%)	54 (15.6%)	29 (11.0%)
2		11 (1.8%)	6 (1.7%)	5 (1.9%)
3		9 (1.5%)	5 (1.4%)	4 (1.5%)
PC-AKI: All KDIGO criteria				
PC-AKI creatinine/urine: No	610	454 (74.4%) [1]	247 (71.2%) [0]	207 (78.7%) [1]
Yes		156 (25.6%)	100 (28.8%)	56 (21.3%)
Highest stage of PC-AKI days 1–3: 0	608	451 (74.2%) [3]	244 (70.9%) [3]	207 (78.4%) [0]
1		52 (8.6%)	35 (10.2%)	17 (6.4%)
2		56 (9.2%)	36 (10.5%)	20 (7.6%)
3		49 (8.1%)	29 (8.4%)	20 (7.6%)

*n* (*p*%) represents the frequency (percentage). Percentages computed by group

[*M*] represents the number of missing

A total of 79 patients (12.9%) were treated with RRT during their ICU stay following CT, with no difference between the groups. The total mortality 180 days after CT was 191 patients (31.2%). Most deaths (139, 72.7% of total mortality) occurred at either the ICU or while still admitted to the hospital. No significant difference between the groups could be seen (Table 3).

**Table 3:** Mortality and need of renal replacement therapy

	*n*	Combined *n* = 611	Contrast at CT		*p* value
			No contrast *n* = 347	Contrast *n* = 264	
Need of renal replacement therapy (RRT) after CT					
Need of RRT after CT	611	79 (12.9%)	48 (13.8%)	31 (11.7%)	0.47^1^
Mortality					
Died at ICU	611	76 (12.4%)	43 (12.4%)	33 (12.5%)	1.00^1^
Died at hospital (ICU excluded)	611	63 (10.3%)	41 (11.8%)	22 (8.3%)	0.18^1^
Died after discharge within 30 days of CT	611	24 (3.9%)	18 (5.2%)	6 (2.3%)	0.091^1^
Died after discharge days 31–90	611	14 (2.3%)	8 (2.3%)	6 (2.3%)	1.00^1^
Died after discharge days 91–180	611	14 (2.3%)	11 (3.2%)	3 (1.1%)	0.11^1^

*n* (*p*%) represents the frequency (percentage). Percentages computed by group

[*M*] represents the number of missing

Plasma creatinine on day of CT was found to have the strongest association with the development of AKI. The second strongest association was male sex. The only other variable with a significant association was MAP on day of CT (Table 4). For long-term plasma creatinine, the strongest associated risk factor was baseline plasma creatinine (*χ*^2^ = 98.1, *p* ≤ 0.001), followed by plasma creatinine on day of CT (*χ*^2^ = 29.3, *p* ≤ 0.001), and CKD (*χ*^2^ = 4.6, *p* = 0.03). No other risk factor had a significant impact on long-term plasma creatinine. PC-AKI had two significant risk factors: the patient’s MAP on day of CT (*χ*^2^ = 19.3, *p* ≤ 0.001), and plasma creatinine on day of CT (*χ*^2^ = 18.7, *p* ≤ 0.001).

**Table 4:** Test of significance for the multivariable model of association between iodine contrast media at CT and acute kidney injury

	Chi-square	df	*p*
Contrast at CT	**0.01**	**1**	**0.903**
Sex	26.06	1	< 0.001
Age	5.77	3	0.123
* Nonlinear*	4.54	2	0.104
Simplified Acute Physiology Score 3	1.39	3	0.707
* Nonlinear*	1.34	2	0.511
Chronic kidney disease	0.79	1	0.374
Diabetes mellitus	0.02	1	0.898
Hypertension	0.01	1	0.933
Plasma creatinine at day of CT	132.73	4	< 0.001
* Nonlinear*	58.81	3	< 0.001
Mean arterial pressure at day of CT	9.62	3	0.022
* Nonlinear*	0.01	2	0.993
Use of renal-affecting drugs	3.03	1	0.082
Use of vasoactive drugs	1.61	1	0.204
Total nonlinear	61.88	9	< 0.001
Total	174.23	20	< 0.001

## Discussion

The main findings of this study are that ICM used for CT was not significantly associated with an increased incidence of AKI or persisting decline in renal function in critically ill patients. Furthermore, this study indicates that, for corresponding plasma creatinine levels, the risk of developing AKI is the same regardless of ICM exposure. This further strengthens previous results that the potential nephrotoxic effects of ICM, as currently used for CT enhancement, are not clinically meaningful [8, 9]. Still, the results indicate that prior renal function is considered when deciding if ICM is to be used for CT enhancement, since the group not receiving ICM had higher plasma creatinine on day of CT and a higher prevalence of CKD. The finding that ICM is not associated with an increase in plasma creatinine levels 3 months after exposure contributes new data to the field. Long-term data for ICM-related renal impairment has rarely been reported, with few studies following patients for more than 72 h [4] (Fig. 2).

The AKI incidence of 41.9% found here is like that seen in the FINNAKI study, but lower than the AKI-EPI study’s 57.3% [3, 23]. All three studies used the complete KDIGO criteria, including urine output, to diagnose AKI. Since several sets of criteria have existed over time and numerous studies have not used the urine output criterion, it is difficult to establish an exact incidence of AKI in critically ill patients [24].

PC-AKI was used as a sensitivity analysis and corroborated the finding using the AKI criteria, since the incidence of PC-AKI did not differ depending on if a patient received ICM or not. This is in line with previous findings in both the critically ill and other patient cohorts [8, 9, 13, 25]. There was a substantial difference in the incidence of PC-AKI depending on if the urine output criterion was included or not. Most studies on AKI and PC-AKI are performed on non-critically ill patients, where urine output is not measured, and therefore only use creatinine-based criteria [26]. Not using the urine output criterion is a limitation since oliguria lasting more than 12 h is associated with worse outcomes, independent of any creatinine elevations [27]. Furthermore, decreased urine output can also reveal AKI earlier than creatinine levels, and there is a risk of missing AKI diagnosis if the urine output criterion is not used [28].

No significant differences in the need for RRT or mortality were seen between patients receiving ICM or not. This is in line with a study in a non-ICU cohort that was unable to show an association between ICM exposure and mortality or the need for RRT [29].

**Fig. 3:**
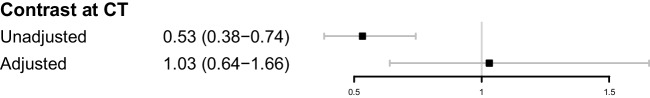
Forest plot of unadjusted and adjusted models for the association between ICM at CT and AKI

3An additional aspect of the data is the steep rise in the risk of developing AKI plateaus at a plasma creatinine value of around 200 µmol/L on day of CT (Fig. 3). Due to reduced statistical power at plasma creatinine levels above 200 µmol/L, the uncertainty in the results is higher, but the attenuation might be due to background fluctuations in plasma creatinine. A substantial proportion of patients have changes of 25% or more in plasma creatinine without any outside influence [15, 16]. This was also seen in the current study, where more than one-fifth of all patients had an increase of 25% or more compared with on day of CT, regardless of ICM exposure. This indicates that the studied patient group does not have stable plasma creatinine values. This fluctuation makes an absolute increase of 26.5 µmol/L more likely the more impaired a patient’s renal function is. The background fluctuation of plasma creatinine also necessitates the use of a control group when studying risk factors for AKI.

The risk factor most strongly associated with developing AKI was plasma creatinine level on day of CT. This is in line with findings regarding AKI and PC-AKI that indicate renal function as a main risk factor for AKI development [3, 30, 31]. Due to the study design with baseline plasma creatinine values extracted 3 months before CT, an elevated plasma creatinine value on day of CT may result in a patient fulfilling the AKI criteria before an exam, which it did for 25.4% of the patients who received ICM and 41.8% for those who did not. If the elevation in plasma creatinine level persisted after CT, the patient was classified as having AKI. Plasma creatinine levels on day of CT were adjusted for in the logistic regression model and no difference in risk of developing AKI could be seen between the groups at corresponding plasma creatinine levels on day of CT. When analyses were performed on PC-AKI, which instead used the change in plasma creatinine from levels just prior to CT, the strength of association between plasma creatinine value on day of CT and risk of developing PC-AKI was not as strong as it was for AKI, although still significant.

Additional risk factors of significance for renal function include MAP on day of CT and, for long-term renal function, if the patient had CKD or not. Since the renal medulla is always on the verge of hypoxia, a decrease in MAP may reduce renal blood flow too far and thus cause AKI [32]. A reduced MAP could also attenuate the glomerular filtration rate through reduced glomerular pressure. This may increase plasma creatinine to AKI levels [33]. Impaired MAP may also indicate more critical illness. Patients with CKD already have reduced renal capacity and might therefore not be able to recover from additional renal insults as easily as other patients. Several risk factors, such as hypertension, diabetes mellitus, SAPS 3, female sex, and use of renal-affecting drugs, seen in other studies to increase AKI incidence, were not found to have a significant impact here [3, 34]. Sepsis, hypovolemia, and major surgery are conditions commonly associated with AKI in intensive care patients [3, 35].

This study has several limitations. First, 37.3% of the patients did not have any baseline plasma creatinine value available. Instead, an estimated baseline was used with an eGFR of 75 mL/min/1.73 m^2^ and a creatinine value was calculated with the MDRD formula. This method is recommended when lacking data on baseline renal function, even though it is imperfect [34]. Since the average eGFR for the studied population, excluding the estimated baseline values, was 74 mL/min/1.73 m^2^, the method tends towards being conservative, thus decreasing the incidence of AKI. However, a difference of 1 mL/min/1.73 m^2^ is clinically negligible and should not affect the conclusions, especially since there was no difference between the groups in the incidence of the estimated baseline value.

A common critique of observational studies in the field of PC-AKI is selection bias. Since patients with healthier kidneys are more likely to receive ICM, and due to their healthier kidneys, they are less likely to develop AKI of any kind. The data clearly demonstrate that, in clinical practice, ICM is avoided in patients with severe reductions in glomerular filtration rate. Guidelines prescribing caution with administering ICM to patients with reduced renal function were used at the clinics included in this study. Consequently, the number of patients with major increases in plasma creatinine who receive ICM is low, leading to a limited possibility to detect significant associations between ICM and AKI in this patient group. However, even in the subjects with plasma creatinine above 300 µmol/L, no tendency for increased odds of AKI due to ICM was noted.

A difference in plasma creatinine levels was seen both at baseline and at CT between the group that received ICM and the group that did not. The patients not receiving ICM also had a significantly higher incidence of fulfilling AKI criteria on day of CT versus their baseline renal function and had four times the prevalence of CKD compared with the group receiving ICM. There was also an unadjusted risk difference where patients receiving ICM had an odds ratio of 0.53 to develop AKI compared with those not receiving ICM. Altogether, patients with severely affected renal function prior to CT were probably less likely to receive ICM. This reduces the possibility of drawing firm conclusions regarding patients with high plasma creatinine levels on day of CT, though results are more reliable for patients with no, minor, or moderate renal impairment on day of CT.

Another limitation was that some patients had repeated CTs within 72 h. Though only the first CT was used for the analysis, a patient who first had an unenhanced CT and then within 72 h had an ICM-enhanced CT might theoretically have results affected by the second CT. The result from the performed sensitivity analysis where these patients were excluded did however not differ from the main analysis.

Urine output was documented once daily and then divided by body weight and 24 to get hourly output. This is one of few studies to include the urine output criterion in the diagnosis of AKI, but the method can only be used to diagnose AKI stage 2 or stage 3. To diagnose stage 1 urine output measurements would have to be recorded at least twice daily.

The generalisability of this study benefits from its bi-centre design, encompassing general ICUs at both a university hospital and a smaller county hospital. Furthermore, all patients who were referred for a CT during the study period were eligible for inclusion, with no patient category or diagnosis excluded. However, external validity is affected since only 36% of the CTs and 8.4% of the total patients treated at the ICUs during the study period were included in the analysis. The three most common reasons for exclusion were ICU treatment for < 48 h, RRT before CT, and missing plasma creatinine values (Fig. 1).

## Conclusions

In this retrospective cohort study of 611 critically ill subjects, administration of ICM was not associated with a long-term (> 3 months) decrease in renal function. Furthermore, ICM did not create an increased risk of AKI, based on changes in plasma creatinine and urine output. This indicates that any nephrotoxic effect of ICM as currently used for CT in critically ill patients is weak and transient. These findings may be considered when weighing the risks and benefits of performing ICM-enhanced radiology in critically ill patients.

### Supplementary Information

Below is the link to the electronic supplementary material.

Supplementary file1 (PDF 283 KB)

## Abbreviations

AKI: Acute kidney injury
CKD: Chronic kidney disease
CT: Computerised tomography
eGFR: Estimated glomerular filtration rate
ICM: Iodine contrast media
ICU: Intensive care unit
KDIGO: Kidney Disease Improve Global Outcome
MAP: Mean arterial pressure
MDRD: Modification of Diet in Renal Disease
PaO_2_/FiO_2_: Partial pressure of arterial oxygen to fractional inspired oxygen
PC-AKI: Post-contrast acute kidney injury
RRT: Renal replacement therapy
SAPS 3: Simplified Acute Physiology Score 3

## Declarations

### Guarantor

The scientific guarantor of this publication is Robert Frithiof.

### Statistics and biometry

Johan Westerbergh is working as a biostatistician at Uppsala Clinical Research Center. He is one of the co-authors.

### Study subjects or cohorts overlap

Parts of the data have been presented as a poster at the European Symposium on Urogenital Radiology 2022 as well as at th European Congress of Radiology 2023.

### Methodology

• retrospective

• observational

• multicentre study
