# Supplementary material for: Acute and long-term renal effects after iodine contrast media–enhanced computerised tomography in the critically ill—a retrospective bi-centre cohort study
Source: Eur Radiol. 2023 Sep 2;34(3):1736–45. doi: 10.1007/s00330-023-10059-7 (PMC10873227; doi:10.1007/s00330-023-10059-7)
Supplement: Supplementary file 1 — Supplementary file1 (PDF 283 KB) [file 330_2023_10059_MOESM1_ESM.pdf]

Supplement A – Details regarding methods, statistical analyses, and models

Background data included age, sex, and weight. Clinical history consisted of chronic heart failure, CKD, diabetes mellitus, hypertension, and liver failure. The main reason for ICU admission was categorised as either cardiac arrest, circulatory failure, infection, respiratory failure, trauma, or other. Acute heart failure or acute liver failure that occurred during the ICU stay was documented and defined as either rapid onset or worsening of earlier symptoms. Simplified Acute Physiology Score 3 (SAPS 3) was recorded [1]. Details regarding CT included if ICM was used and, if so, which dose, type, and concentration.

Treatments received included any mechanical respiratory treatments (divided into invasive and non-invasive), antibiotics, vasoactive drugs, and drugs affecting the kidneys. Atropine, dopamine, dobutamine, ephedrine, epinephrine, norepinephrine, and phenylephrine were considered vasoactive drugs. Acyclovir, aminoglycosides, amphotericin B, angiotensin II-converting enzyme inhibitors, angiotensin II receptor blockers, any diuretics, any nonsteroidal anti-inflammatory drugs, ciclosporins, colistin, gadolinium, hydroxyl-ethyl-starch, mannitol, tacrolimus, and vancomycin were considered drugs affecting the kidneys. Any use of these drugs within 24 hours pre- or post-CT, except diuretics, where the time limit was extended to 48 hours post-CT, was recorded. Treatment with N-acetylcysteine before CT was also noted.

Physiological parameters included partial pressure of arterial oxygen to fractional inspired oxygen (PaO_2/FiO_2), lowest mean arterial pressure (MAP) during ICU stay, and MAP before CT (recorded as close in time to CT as possible). The highest lactate level and the lowest haemoglobin concentration during the ICU stay were recorded.

Fluid intake during day of CT was measured, as well as fluid balance on day of CT and the following day. Hourly urine excretion in relation to body weight was measured at 12 hours before and 12 hours after CT. Total urine output was measured once per day for the three days following CT. The same urine output measurements were used to define both AKI and PC-AKI.

A need for RRT during the ICU stay post-CT, mortality during ICU stay, during hospital stay, and on days 30, 90, and 180 post-CT were also documented.

The risk of AKI was analysed using two models, one unadjusted model including only contrast at CT and one model adjusting for age, sex, SAPS 3, CKD, diabetes, hypertension, creatinine at CT, MAP, use of renal-affecting drugs, and use of vasoactive drugs. All continuous variables (age, SAPS 3, creatinine at CT, MAP) were fitted using splines with four knots for all variables except plasma creatinine, which was fitted with five knots.

The interaction effect between contrast at CT and creatinine at CT was also examined using two models, one unadjusted including only these two variables and the interaction* between them, and one adjusted model adjusting for the variables stated in the previous paragraph.

Long-term creatinine was analysed using baseline creatinine and if contrast was used at any CT for that patient. An adjusted model was also used, adjusting for age, sex, SAPS 3, CKD, diabetes, hypertension, highest measured creatinine, lowest measured MAP, days at ICU, if the patient had any AKI, use of renal-affecting drugs, and use of vasoactive drugs.

**Only the linear part of the spline was used for the interaction.*

Supplement B – Additional results, figures, and tables

Table S1 – Type and dose of ICM used.

Table S2 – Examined body part, several patients had more than one body part examined during the same CT.

Figure S1 – Predicted risk of developing PC-AKI by plasma creatinine value (µmol/L) on day of CT for ICM vs. no ICM, unadjusted model (adjusted model yielded similar results). The figure shows the patients' plasma creatinine value on day of CT on the X axis and the predicted risk of developing AKI on the Y axis, the shaded area represents the 95% confidence bands for the lines. The left panel shows PC-AKI defined using only the two creatinine-based criteria, the right panel shows PC-AKI defined using both creatinine and urine output criteria.

Approximately one fifth of all patients had a 25% increase in plasma creatinine value during the 72 hours following CT compared with the value on day of CT. Furthermore, 38 patients (11.0%) in the

group not receiving ICM and 34 (12.9%) of patients receiving ICM had a plasma creatinine increase of at least 25% during the 72 hours following CT (Table S3).

Table S3 – Increases of at least 25% either during the 72 hours following CT or compared with day of CT.

Those in the ICM-receiving group were more frequently treated with N-acetylcysteine and received a larger volume of fluids on day of CT (Table S4). The group that developed AKI had a higher net balance of fluids on day of CT but did not receive more fluids or have a higher frequency of treatment with N-acetylcysteine (Table S5). Prophylactic rehydration is recommended for at-risk patients before undergoing CT, even though no preventative effect has been found in randomized controlled studies [2-4]. In this study, no difference in received amounts of fluid was detected between patients who developed AKI and those who did not. Still, patients who developed AKI had a higher net fluid balance on day of CT, though that difference had disappeared the following day. Fluid overload, even during limited time periods, is known to worsen outcome for critically ill patients, and might also decrease the chances of renal recovery [5]. Subjects who developed AKI and those who did not had an equal frequency of treatment with N-acetylcysteine, another drug used as prophylaxis. This is consistent with a previous randomized controlled study, which did not find a protective effect of N-acetylcysteine [6].

Table S4 – Fluid treatment and balance and treatment with N-acetylcysteine, divided into contrast or no contrast, volume received, and net balance.

Table S5 – Fluid treatment and balance and treatment with N-acetylcysteine, divided into development of AKI or not within 3 days of CT, volume received and net balance.

References

1. Moreno RP, Metnitz PG, Almeida E, Jordan B, Bauer P, Campos RA, et al. SAPS 3--From evaluation of the patient to evaluation of the intensive care unit. Part 2: Development of a prognostic model for hospital mortality at ICU admission. Intensive Care Med. 2005;31(10):1345-55.
2. Nijssen EC, Rennenberg RJ, Nelemans PJ, Essers BA, Janssen MM, Vermeeren MA, et al. Prophylactic hydration to protect renal function from intravascular iodinated contrast material in patients at high risk of contrast-induced nephropathy (AMACING): a prospective, randomised, phase 3, controlled, open-label, non-inferiority trial. Lancet. 2017;389(10076):1312-22.
3. ACR. ACR Manual on Contrast Media. American College of Radiology: American College of Radiology; 2020. Report No.: 978-1-55903-012-0.
4. ESUR. ESUR Guidelines on Contrast Agents. European Society of Urogenital Radiology; 2018 2018-03.

5. Bouchard J, Mehta RL. Fluid accumulation and acute kidney injury: consequence or cause. Curr Opin Crit Care. 2009;15(6):509-13.

6. Brueck M, Cengiz H, Hoeltgen R, Wieczorek M, Boedeker RH, Scheibelhut C, et al. Usefulness of N-acetylcysteine or ascorbic acid versus placebo to prevent contrast-induced acute kidney injury in patients undergoing elective cardiac catheterization: a single-center, prospective, randomized, double-blind, placebo-controlled trial. J Invasive Cardiol. 2013;25(6):276-83.

Supplementary figure

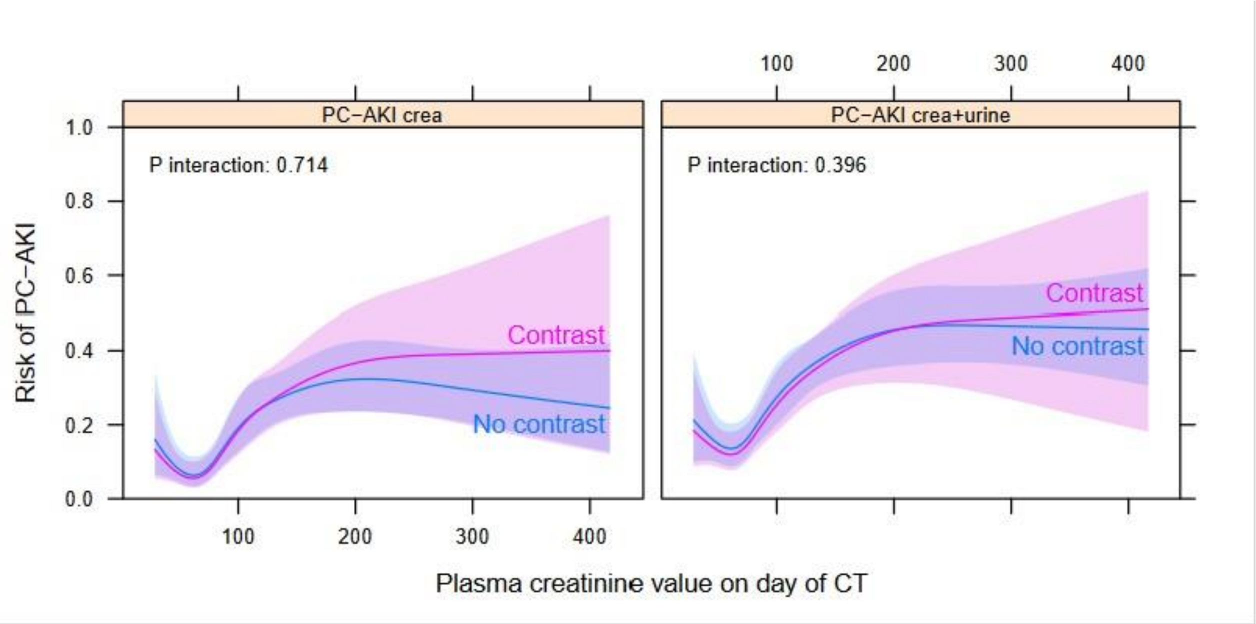

Supplementary table 1

	n	
	611	
**Contrast at CT**	611	
No		347 (56.8)
Yes		264 (43.2%)
**Type of iodine contrast media (ICM)**	259 [5]	
Iohexol 140 mg I/ml		1 (0.4%)
Iohexol 180 mg I/ml		3 (1.2 %)
Iohexol 240 mg I/ml		2 (0.8%)
Iohexol 300 mg I/ml		1 (0.4%)
Iohexol 350 mg I/ml		208 (80.0%)
Iohexol unknown concentration		42 (16.2%)
Ioversol 350 mg I/ml		1 (0.4%)
Iomeprol 400 mg I/ml		1 (0.4%)
**Dose of ICM (ml/kg)**	215	1.0 (0.8 – 1.3) [49]

m (a – b) represents median (IQR).
n (p%) represent frequency (percentage). Percentages computed by group.
[M] represents number of missing.

Supplementary table 2

**Body part**	**Contrast at CT**	
	No contrast	Contrast
Abdomen	82 (18.8%)	115 (28.7%)
Angiography	0	5 (1.2 %)
Head	172 (39.4%)	56 (14%)
Skeletal	24 (5.5%)	13 (3.2%)
Thorax	149 (34.1%)	178 (44.4%)
Trauma	0	5 (1.2%)
Urinary tract	2 (0.5%)	1 (0.2%)
Other	7 (1.6%)	28 (7%)

Supplementary table 3

	n	Combined	Contrast at CT	
			No contrast	Contrast
25% increase in creatinine within 72 h	611	72 (11.8%)	38 (11.0%)	34 (12.9%)
25% increase in creatinine within 72 h compared to day of CT	611	126 (20.6%)	73 (21.0%)	53 (20.1%)

n (p%) represent frequency (percentage). Percentages computed by group.

Supplementary table 4

	n	Combined	Contrast at CT: No contrast	Contrast at CT: Contrast	P-value
N-acetylcysteine treatment	611	35 (5.7%)	5 (1.4%)	30 (11.4%)	<0.001[1]
Volume received on day of CT	455	3270.0 (2656.0 -- 4613.5) [156]	3144.0 (2520.8 -- 4425.0) [85]	3483.0 (2772.0 -- 4775.0) [71]	0.034[2]
Net balance day of CT	469	1810.0 (179.0 -- 4474.0) [142]	1874.0 (113.0 -- 4600.0) [86]	1765.5 (270.0 -- 4245.0) [56]	0.96[2]
Net balance day 1	452	1213.5 (-122.8 -- 3422.5) [159]	1200.0 (-42.5 -- 3916.0) [96]	1214.0 (-307.0 -- 2938.0) [63]	0.23[2]

m (a – b) represents median (IQR).
n (p%) represent frequency (percentage). Percentages computed by group.
[M] represents number of missing.

Tests used: 1 Fisher's exact test; 2 Wilcoxon test.

Supplementary table 5

	n	Combined	AKI within 3 days of CT		P-value
			No	Yes	
N-acetylcysteine treatment	611	35 (5.7%)	22 (6.2%)	13 (5.1%)	0.60[1]
Volume received on day of CT	455	3270.0 (2656.0 -- 4613.5) [156]	3190.0 (2660.0 -- 4296.0) [80]	3409.5 (2636.0 -- 4962.8) [76]	0.40[2]
Net balance day of CT	469	1810.0 (179.0 -- 4474.0) [142]	1520.0 (180.0 -- 3860.8) [73]	2642.0 (183.0 -- 4937.5) [69]	0.047[2]
Net balance day 1	452	1213.5 (-122.8 -- 3422.5) [159]	1227.0 (8.2 -- 3239.2) [83]	1164.0 (-440.5 -- 4410.0) [76]	0.58[2]

m (a – b) represents median (IQR).

n (p%) represent frequency (percentage). Percentages computed by group.

[M] represents number of missing.

Tests used: 1 Fisher’s exact test; 2 Wilcoxon test.
